# RETRACTED: Correction to: ‘Reef fishes can recognize bleached habitat during settlement: sea anemone bleaching alters anemonefish host selection’

**DOI:** 10.1098/rspb.2022.1217

**Published:** 2022-07-13

**Authors:** Anna Scott, Danielle L. Dixson

*Proc. R. Soc. B* **283**, 20152694 (Published online 25 May 2016) (https://doi.org/10.1098/rspb.2015.2694)

The published article [1] requires a correction. First, the experimental dates listed in the materials and methods section were incorrectly reported. The correct experimental timeframe during which we recorded host selection using two flumes was 5 October–7 November 2014. Of note, this error does not impact or change any of the results or conclusions of our work.

Second, a summary table rather than the raw dataset was uploaded to a public repository. This has been corrected, and the raw data are available at https://doi.org/10.5281/zenodo.6565204.

Additionally, we would like to clarify that the strong treatment effects found in this experiment are likely the result of a combination of factors including, but not limited to, the ecology of the focal species, the flume apparatus, the chemical comparisons being tested as well as the concentration of the chemical cues tested. The chemical cue concentration is likely higher than the organisms would experience in nature, and therefore likely a supernormal stimulus. Simply put, supernormal stimuli are bigger and more intense than normal, and elicit a larger than normal response from the animal [2]. Here, the naturally occurring olfactory cues indicate habitat; the heightened preference when the stimulus is offered at an intense concentration follows this behavioural pattern. The research presented in this study purposefully used strong chemical cues to determine *if* chemical cues are used in habitat selection, rather than determining a detection threshold for this species of a concentration gradient.
